# Aldose reductase participates in the downregulation of T cell functions due to suppressor macrophages

**DOI:** 10.1038/srep21093

**Published:** 2016-02-12

**Authors:** Toshiaki Shimizu, Yutaka Tatano, Haruaki Tomioka

**Affiliations:** 1 Department of Nutritional Sciences, Yasuda Women’s University, Hiroshima 731-0153, Japan; 2 Department of Pharmaceutical Sciences, International University of Health and Welfare, Ohtawara 324-8501, Japan; 3 Department of Basic Medical Sciences for Nursing, Yasuda Women’s University, Hiroshima 731-0153, Japan; 4 Department of Microbiology and Immunology, Shimane University School of Medicine, Izumo 693-8501, Japan

## Abstract

The cell-to-cell contact of T lymphocytes with immunosuppressive macrophages causes marked changes in the tyrosine phosphorylation of some cytosolic proteins of T cells. By phosphoproteome analysis, we identified a 36-kDa protein as aldose reductase (AR). The AR expression in T cells was not changed by TCR stimulation or due to cell-to-cell transmission of suppressor signals from immunosuppressive macrophages. Therefore, AR phosphorylation/dephosphorylation is essential for the transduction of TCR-mediated T-cell stimulatory signals, and moreover plays important roles for the cross-talk of immunosuppressive macrophage-derived suppressor signals with the signaling pathways for T-cell activation. Moreover, AR played important roles in the upregulation of ERK1/2-mediated signaling pathways in T lymphocytes. Notably, the enzymatic activity of AR was not required for its signaling action. Taken together, it is concluded that AR mediates intracellular transmission of the suppressor signal of immunosuppressive macrophages toward downstream ERK1/2 pathways, possibly through its direct interaction with acceptor proteins.

During the course of mycobacteriosis in humans and experimental animals, the generation of immunosuppressive macrophages (MΦs) is frequently encountered^1^,^2^. These MΦs suppress T cell functions, including their proliferative response and Th1 cytokine production due to T cell receptor (TCR) ligation, causing the suppression of cellular immunity in the advanced stages of infection^2^. Previously, we found that immunosuppressive MΦs were induced in the spleens of mice infected with mycobacterial pathogens, such as the *Mycobacterium avium* complex (MAC), and that such an immunosuppressive MΦ (designated MAC-MΦ) population displayed potent suppressor activity against proliferative response of T cells to TCR signaling and Con A stimulation^3^,^4^. Suppressive signals of MAC-MΦs were partly transmitted via humoral effectors, including reactive nitrogen intermediates (RNIs), TGF-β, and prostaglandin E, similar to other kinds of suppressor MΦs, such as those generated in tumor-bearing hosts (tumor-associated MΦs) and induced by mycobacterial (*M. bovis* BCG), protozoal, and helminth infections^2^,^5^,^6^,^7^. In this context, the M2-type MΦs expressing an IL-12^low^, IL-23^low^, IL-10^high^ phenotype share functional properties characteristic of suppressor macrophages^2^,^8^,^9^. Indeed, immature myeloid suppressor cells are known to have functional properties and a transcriptional profile related to M2 MΦs^10^. The M2-type MΦs also produce Th2 cytokines, such as IL-10, as immunosuppressive mediators^2^,^8^,^9^. In this context, we recently found that a novel MΦ population, which is functionally distinguishable from ordinary M1 and M2 MΦ subsets and possesses unique phenotypes (IL-12^+^, IL-1β^high^, IL-6^+^, TNF-α^+^, nitric oxide synthase 2 (NOS2)^+^, CCR7^high^, IL-10^high^, arginase-1^−^, mannose receptor^low^, Ym1^high^, Fizz^low^, and CD163^high^), up-regulates Th17 cell expansion through the action of IL-6 and TGF-β but not IL-21 and IL-23^11^.

In the case of MAC-MΦs, we found that cell contact of MAC-MΦs with target T cells is required to effectively induce their suppressor activity^12^. The suppressor signals of MAC-MΦs, which are transmitted to the target T cells via cell contact, principally cross-talk with the early signaling events before the activation of protein kinase C (PKC) and/or intracellular calcium mobilization^12^. Indeed, the pre-cultivation of T cells with MAC-MΦs, facilitating cell-to-cell contact, reduced anti-CD3 Ab-induced mitogenesis but not the phorbol myristate acetate/calcium ionophore A23187-elicited proliferation of T cells^12^. It was also found that a B7-1-like molecule (B7-1LM) on MAC-MΦs, but not B7-2, ICAM-1, nor VCAM-1 molecule, plays important roles in the transmission of suppressor signals from MAC-MΦs to target T cells through cell-to-cell interaction^13^. The mAb-blocking of CTLA-4 on target T cells did not reduce the suppressor activity of MAC-MΦs, suggesting the role of a putative molecule on target T cells other than CTLA-4 as a receptor for B7-1LM of MAC-MΦs^13^. In this context, the co-cultivation of T cells with MAC-MΦs caused marked changes in the profiles of the tyrosine (Tyr) phosphorylation of several cytosolic proteins with molecular weights (MWs) of around 35 kDa^12^. Tyr residues of these proteins were dephosphorylated in response to suppressor signals from MAC-MΦs.

In the present study, we attempted to identify these cytosolic proteins, and found that one of these proteins (36-kDa protein) corresponds to aldose reductase (AR), a member of the aldo-keto reductase superfamily, which catalyzes the reduction of a wide range of aldehydes, including glucose^14^. Interestingly, AR is known to play important roles in intracellular signal transduction involving phospholipase C (PLC), PKC, MAP kinase (MAPK), and NF-κB pathways, leading to inflammatory reactions and the expression of adhesion molecules^15^,^16^,^17^,^18^. Therefore, we examined detailed profiles of the participation of AR in the intracellular transmission of immunosuppressive MΦ-derived suppressor signals in the target T cells.

## Results

### Cell-to-cell contact of T cells with suppressor MΦs decreases the levels of Tyr phosphorylation of AR of target T cells

**Figure 1:**
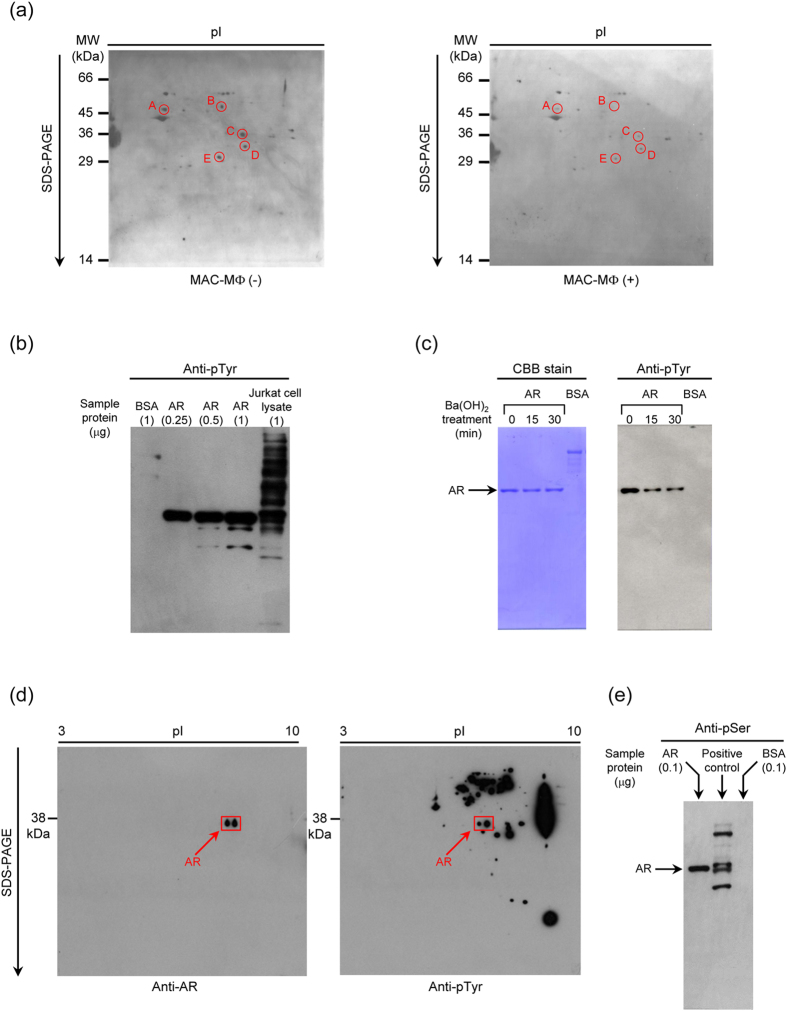
Evidence that AR is phosphorylated at tyrosine residues in target T cells which received MAC-MΦ-derived suppressor signals via cell-to-cell contact. (**a**) Immunoblotting of cellular proteins with pTyr residues in cell lysates of T cells which had been cultured with or without cell contact with MAC-MΦs. The five spots (No. A to E) showed significantly decreased Tyr phosphorylation levels after 23-h cultivation with MAC-MΦs allowing cell-to-cell contact. (**b**) Immunoblotting of recombinant preparations of human AR, which were expressed in insect cells for pTyr. Jurkat cell lysate and BSA were used as positive and negative controls, respectively. The AR molecules were found to be phosphorylated at certain Tyr residues. (**c**) Decrease in reactivity of the AR molecules to anti-pTyr Ab by treatment with Ba(OH)_2_. The AR molecules were found to have pTyr residues which could be chemically dephosphorylated. (**d**) Profile of Tyr phosphorylation in AR molecules detectable in a cell lysate of mouse splenic T cells. Cell lysate proteins of murine T cells were developed by two-dimensional electrophoresis and immunoblotted using anti-AR and anti-pTyr Abs. Mouse T cells contained two types of AR with differential electronic charges, both of which had pTyr residues. (**e**) Positive reaction of an anti-pSer Ab with the human AR molecules. The AR preparation was immunoblotted with anti-pSer Ab using the control proteins having pSer residues (Calbiochem) and BSA, as positive and negative controls, respectively. The human AR molecules were found to possess not only pTyr but also pSer residues.

Splenic T cells were cultured with MAC-MΦs allowing cell-to-cell contact for 23 h, and the resultant T cells (non-adherent cells) were collected. In the present study, we usually used nylon wool column non-adherent T cells without fractionating to CD4^+^ and CD8^+^ T cell subsets. The major T cell population, the TCR-signaling-induced mitogenic response of which is suppressed by MAC-MΦs, is thought to comprise CD4^+^ T cells, since the co-cultivation of test T cells with MAC-MΦs reduced the CD4^+^ T cell population but increased CD8^+^ T cells (unpublished observation). Cell lysate of the T cells was prepared and subjected to two-dimensional electrophoresis followed by Western blotting using anti-phosphorylated Tyr (pTyr) mAb. As indicated in Fig. 1a, five spots (No. A to E) showed a significantly decreased intensity of pTyr in T cells after co-cultivation with suppressor MΦs allowing cell contact, compared to T cells cultured alone. The relative intensity of individual spots after co-cultivation with MAC-MΦs was as follows: Spot A, 0.17; Spot B, 0.08; Spot C, 0.15; Spot D, 0.24; Spot E, 0.35 when the intensity of T cells cultured alone was set to 1.0. MALDI-TOF mass analysis of the 36-kDa protein (spot No. C) indicated that this protein corresponds to AR on the basis of masses of several peptide fragments (Table 1).

AR is a member of the aldo-keto reductase superfamily and catalyzes the reduction of a wide range of aldehydes, including glucose and other saturated and unsaturated aldehydes, especially lipid aldehydes such as 4-hydroxynonenal^14^,^19^,^20^. Reduction of glucose by AR generates sorbitol, which is used for fructose synthesis, or as an inert osmolyte in the renal medulla^21^. Therefore, AR is a critical participant in osmoregulation and increased sorbitol generation, and accumulation is linked to the development of secondary diabetic complications^14^. Notably, it is known that AR participates in signaling pathways in certain types of cell, such as MΦs, microglial cells, vascular smooth muscle cells, and endothelial cells, and up-regulates the production of inflammatory cytokines, enzymes, and adhesion molecules, via signaling axes including PKC, IκB kinase (IKK)/NF-κB, Rho kinase/MKK/MAPK, and AP-1 signaling axes^15^,^16^,^17^,^18^,^22^,^23^,^24^,^25^. The AR-mediated generation of 4-hydroxynonenal also causes the increased phosphorylation of cAMP response element-binding protein and consequent promotion of Arg1 expression, thereby resulting in microglial/macrophage polarization^26^. We therefore focused on the participation of AR in the intracellular transduction of suppressor MΦ signals in the target T cells. Firstly, we examined whether or not AR acts as a substrate for Tyr kinase and Tyr phosphatase *in vitro*. The recombinant AR expressed in insect cells, which had been transfected with human AR gene-carrying baculovirus vector, was subjected to Western blotting analysis using anti-pTyr mAb. As shown in Fig. 1b, the AR band was strongly stained with anti-pTyr mAb, while the band corresponding to BSA was not stained for pTyr, indicating that the used AR preparation was phosphorylated at certain Tyr residues. Secondly, when the recombinant AR preparation was dephosphorylated by Ba(OH)_2_ treatment, the reactivity to anti-pTyr mAb was time-dependently decreased during the course of dephosphorylation (Fig. 1c). The relative intensity of bands of AR stained with anti-pTyr Ab after Ba(OH)_2_ treatment was as follows: Control (untreated AR), 1.0; 15 min after Ba(OH)_2_ treatment, 0.50; 30 min after Ba(OH)_2_ treatment, 0.48. The intensity of bands of AR stained with CBB distributed within 0.98 to 1.0. Next, we examined the profiles of Tyr phosphorylation in T cells. When the cell lysate of splenic T cells was analyzed by Western blotting for the presence of AR with phosphorylated Tyr residues, two spots of 36-kDa proteins corresponding to AR were detected (Fig. 1d). Notably, the two spots had markedly different intensities of pTyr staining. When the intensity of spots of AR stained with anti-AR Ab or anti-pTyr Ab was analyzed by densitometry, the relative intensity of the right spots was measured as follows: anti-AR Ab staining, 1.18; anti-pTyr Ab staining, 2.52, as compared with those of the left spots fixed to 1.0. A weakly stained pTyr spot was expected to correspond to phosphorylated serine (Ser) or threonine (Thr) residues. This was confirmed by the Western blotting experiments using anti-phosphorylated Ser (pSer) monoclonal antibody (mAb) (Fig. 1e). The recombinant AR preparation was strongly stained with mAb specific for a phosphorylated Ser residue, while the BSA preparation was not stained with this mAb. These findings indicate the following. Firstly, one of the T cell’s cytosolic proteins, which are dephosphorylated in response to MAC-MΦ-originated suppressor signals transmitted to the target T cells via cell-to-cell contact, is AR. Secondly, AR exists in the cytosol of T cells as a constitutively phosphorylated protein in its Tyr and Ser residues, and its pTyr residues can be dephosphorylated under certain conditions.

**Figure 2:**
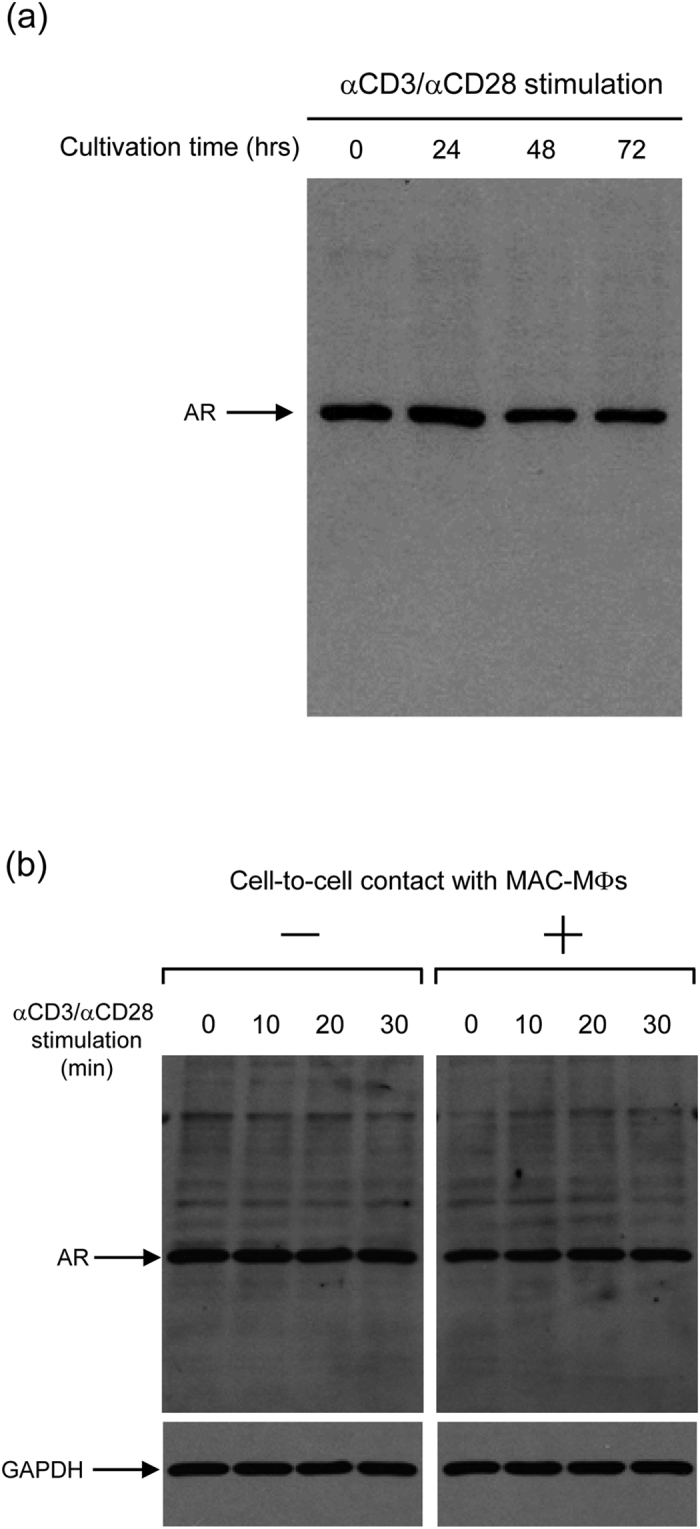
Expression of AR in mouse splenic T cells. (**a**) Profiles of AR expression in T cells before and after stimulation with anti-CD3 and anti-CD28 Abs. The levels of expression of cellular AR in T cells were not changed during cultivation after TCR stimulation. (**b**) Profiles of AR expression in T cells before and after cultivation with MAC-MΦs allowing cell-to-cell contact. The levels of expression of AR in the target T cells was found not to be altered due to cell contact with MAC-MΦs.

As shown in Fig. 2a, Western blotting analysis using anti-AR Ab indicated that the expression of AR by T cells slightly decreased after the stimulation with anti-CD3 mAb in combination with anti-CD28 mAb during time periods of 48- to 72-h cultivation. The relative intensity of the AR bands was changed during cultivation after TCR stimulation as follows: 0-time, 1.0; 24 h, 1.18; 48 h, 0.87; 72 h, 0.86. In addition, when T cells were cultured alone or co-cultured with MAC-MΦs allowing cell-to-cell contact for 23 h and then stimulated with anti-CD3 antibody (Ab) in combination with anti-CD28 Ab, the AR expression in the target T cells did not change during the 30 min after TCR stimulation (Fig. 2b). The relative intensity of the AR bands after TCR stimulation expressed by T cells cultured with or without MAC-MΦs was as follows: cultured without MAC-MΦ: 0-time, 1.0; 10 min, 0.95; 20 min, 0.94; 30 min, 0.90; cultured with MAC-MΦ: 0-time, 1.0; 10 min, 0.95; 20 min, 1.10; 30 min, 1.08. There was no statistical difference between the profiles of AR expression of T cells with or without co-cultivation with MAC-MΦs (*p* = 0.18; Two-way ANOVA). This indicates that suppressor signals from MAC-MΦs did not affect the expression of AR protein in the target T cells. Taken together, it appears that the immunosuppressive signals from the suppressor MΦs dephosphorylate Tyr residues of AR in the target T cells, possibly due to some Tyr phosphatases.

### Roles of AR in signaling pathways for T-cell activation induced by TCR stimulation

**Figure 3:**
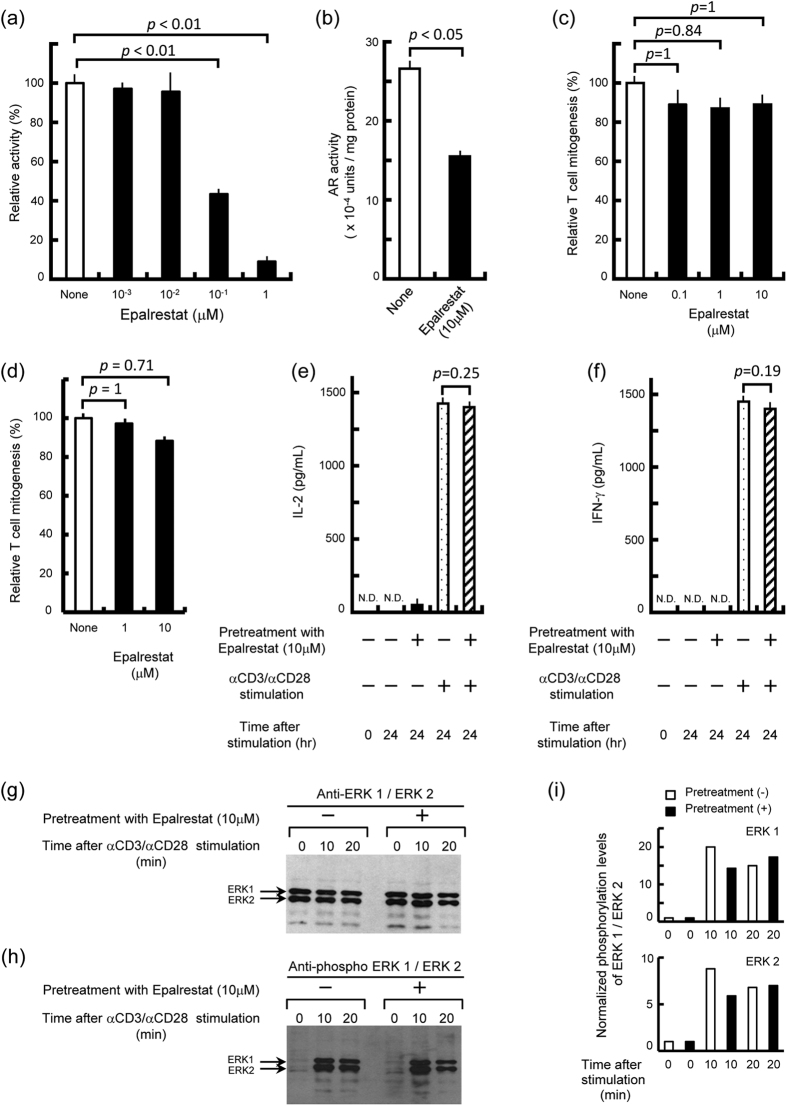
Evidence that enzymatic activity of AR is not essential for signaling pathways of T cells leading to the activation of T-cell functions. (**a**) Inhibitory effects of epalrestat against enzymatic activity of recombinant human AR. (**b**) Effects of epalrestat on the cellular AR activity when D10.G4.1 mouse Th2 cells were treated with 10 μM epalrestat. (**c–f**) Effects of the AR inhibitor epalrestat on the proliferative response (**c, d**), IL-2 production (**e**), and IFN-γ production (**f**) of mouse splenic T cells induced by anti-CD3 and anti-CD28 Abs (**c, e, f**) or Con A (**d**). The inhibition of enzymatic activity of AR did not affect the T-cell proliferative response or IL-2 and IFN-γ expressions responding to TCR stimulation. (**g, h**) Effects of epalrestat on the cellular levels of ERK1/2 expression (**g**) and phosphorylation of ERK1/2 (**h**) in splenic T cells before and after stimulation with anti-CD3 and anti-CD28 Abs. The inhibition of AR activity had no significant influence on the expression and phosphorylation profiles of ERK1/2 in T cells undergoing TCR stimulation. **(i**) Normalized phosphorylation levels of ERK1/2 in T cells before and after TCR stimulation. There was no significant change due to inhibition of the enzymatic activity of AR in the levels of phosphorylation of ERK1/2 per molecule.

AR exhibits its enzymatic activity to reduce lipid aldehydes, such as 4-hydroxynonenal and glutathionyl 4-hydroxynonenal^27^,^28^. It has been demonstrated that pharmacological inhibition and antisense ablation of AR’s activity prevented PLC, PKC, IKKα/β, and NF-κB activation caused by these lipid aldehydes and other stimulating signals that up-regulate the production of reactive oxygen intermediates (ROIs) by MΦs and vascular smooth muscle cells^22^,^23^,^24^. Furthermore, the AR-mediated generation of sorbitol from glucose is known to down-regulate the expression of certain types of microRNA in renal mesangial cells, thereby resulting in the potentiated expression of TGF-β and reduced expression of NF-E2-related factor 2^29^. Based on these findings, we subsequently examined the effects of the AR inhibitor epalrestat on the proliferative response of T cells after TCR stimulation. This approach may be useful to elucidate the molecular mechanisms by which AR participates in the signaling pathways for T-cell activation in response to TCR stimulation. Firstly, epalrestat caused 57 and 91% inhibition (*p* < 0.01; Bonferroni’s multiple *t*-test) of the AR activity at 0.1 and 1 μM, respectively, under the standard conditions for the *in vitro* enzyme assay (Fig. 3a). Next, the D10.G4.1 mouse Th2 cell line (6 × 10^6^ cells) was cultured in 5% FBS-RPMI medium in the presence or absence of 10 μM epalrestat at 37 °C for 24 h. Then, cultured cells were harvested and extracted with 135 mM Na-K phosphate buffer (pH 7.0) by sonication, and the resultant cell lysate was measured for its AR activity. As shown in Fig. 3b, the treatment of test cells with 10 μM epalrestat caused 42% inhibition of cellular AR activity (*p* < 0.05; Mann-Whitney U test). Next, the effects of epalrestat on T cell-functions were examined. As shown in Fig. 3c, T-cell mitogenesis induced by anti-CD3 and anti-CD28 Abs was only slightly affected by the addition of epalrestat, even at 10 μM (11% inhibition; statistically insignificant, *p* = 0.84 to 1.0; Bonferroni’s multiple *t*-test). The same concentration of epalrestat had no cytotoxic effect on T cells in terms of the inhibition of the Con A-induced proliferation of target T cells (cytotoxicity was statistically insignificant, *p* = 0.71 to 1.0; Bonferroni’s multiple *t*-test) (Fig. 3d) and morphological changes of Epalrestat-treated T cells during 72-h cultivation after TCR stimulation (data not shown). This value was much lower than that of the inhibition of cellular AR activity by 10 μM epalrestat (42% inhibition) (Fig. 3b). We also examined the effects of epalrestat-treatment on the production of Th1 cytokines by T cells after stimulation with anti-CD3 and anti-CD28 Abs. As indicated in Fig. 3e, f, the enzymatic inhibition of AR by epalrestat did not significantly affect IL-2 and IFN-γ production by T cells in response to TCR stimulation (*p* = 0.25 and 0.19, respectively; Mann-Whitney U test).

It is known that TCR stimulation by anti-CD3 and anti-CD28 Abs induces the activation of ERK 1/2 in the late signaling pathways^30^. In addition, it has been reported that the over-expression of AR in the AML12 mouse hepatocyte cell line increased the level of phosphorylated ERK1/2 (pERK1/2), resulting in the phosphorylation of peroxisome proliferation-activated receptor α (PPARα), indicating that AR plays an important role in the regulation of hepatic PPARα activation via the phosphorylation of ERK1/2^31^. Therefore, we examined the effects of AR inhibition by epalrestat treatment on the phosphorylation of ERK1/2 in T cells. As shown in Fig. 3g, h, epalrestat failed to significantly reduce the levels of expression and phosphorylation of ERK1/2. Normalization of the band intensity of pERK1/2 for protein expression indicated that there was no significant change due to epalrestat-treatment in the level of phosphorylation of ERK1/2 per molecule (*p* = 0.73 between epalrestat-treatment (+) and epalrestat-treatment (−) for ERK1 and *p* = 0.23 between epalrestat-treatment (+) and epalrestat-treatment (−) for ERK2; Two-way ANOVA) (Fig. 3i). These findings suggest that the enzymatic activity of AR is not required for AR’s action as a mediator of intracellular signal transmitting pathways from TCR to MAPK cascades.

### Cross-talk of the suppressive signals from suppressor MΦs with the signaling pathways in TCR-stimulated T cells

**Figure 4:**
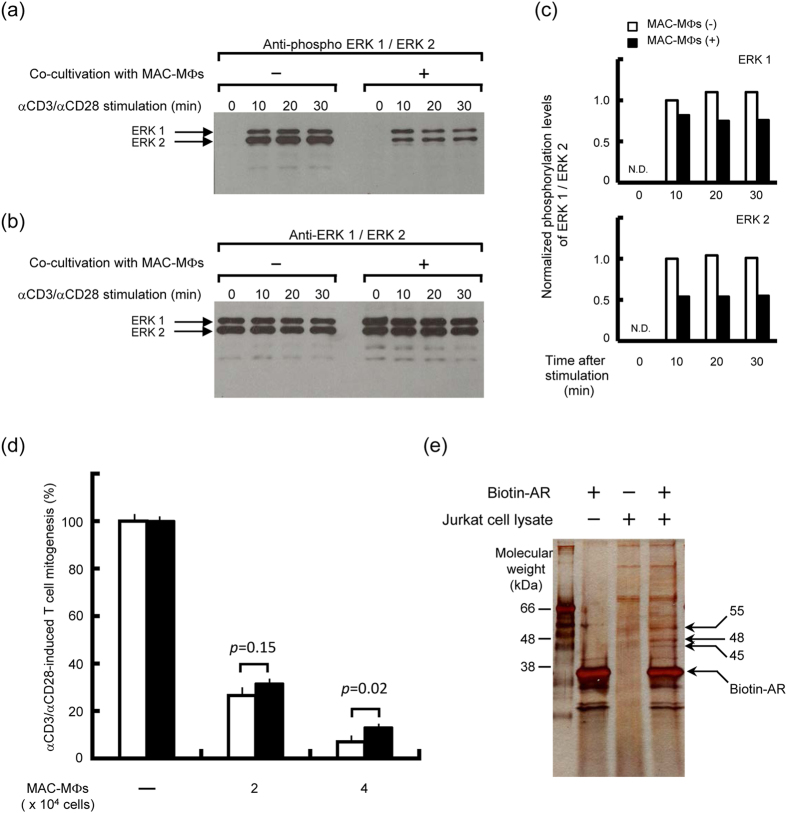
Effects of the suppressor signals from MAC-MΦs via cell-to-cell contact upon activation of ERK1/2 and the proliferative response of T cells induced by TCR stimulation. (**a–c**) Effects of suppressor signals of MAC-MΦs transmitted to the target T cells by co-culturing with MAC-MΦs on phosphorylation of ERK1/2 induced by TCR stimulation. Mouse splenic T cells were co-cultured with MAC-MΦs allowing cell-to-cell contact for 18 h, and thereafter stimulated with anti-CD3 and anti-CD28 Abs. After 10- to 30-min incubation, cell lysates of the resultant T cells were prepared and subjected to immunoblotting using anti-phospho ERK1/2 (**a**) and anti-ERK1/2 (**b**) Abs. The immunosuppressive signal from MAC-MΦs transmitted by cell contact was found to inhibit ERK1/2 activation without affecting the expression of ERK1/2 in the target T cells after TCR stimulation. (**c**) Normalized phosphorylation levels of ERK1/2 in T cells before and after TCR stimulation with or without pre-cultivation of T cells in the presence or absence of MAC-MΦs. (**d**) Effects of epalrestat on MAC-MΦ-mediated suppression of the proliferative response of T cells induced by TCR stimulation. The target T cells had been treated (closed bar) or not treated (open bar) with epalrestat for 24 h before 72-h cultivation after stimulation with anti-CD3 and anti-CD28 Abs in the presence or absence of MAC-MΦs. The inhibition of the enzymatic activity of AR in the target T cells was found not to affect manifestation of the suppressor action of MAC-MΦs. The representative results obtained by repeated experiments are indicated. (**e**) Binding profile of human AR molecules with cellular proteins of T cells using the pull-down assay method.

Next, we examined profiles of cross-talk of MAC-MΦ-originated suppressive signals with the signaling cascades of T cells in the downstream pathways of TCR stimulation (Fig. 4a–c). As shown in Fig. 4a, Western blotting analysis using anti-pERK1/2 mAb demonstrated that T-cell stimulation with anti-CD3 and anti-CD28 Abs caused the phosphorylation of ERK1/2 at 10 to 30 min after TCR stimulation. Co-cultivation of the target T cells with the suppressor MΦs allowing cell-to-cell contact resulted in nearly significant levels of reduction in the phosphorylation levels of ERK1/2 molecules (*p* = 0.08 between MAC-MΦ (+) and MAC-MΦ (−) for ERK1 and *p* = 0.06 between MAC-MΦ (+) and MAC-MΦ (−) for ERK2; Two-way ANOVA (Fig. 4a, c). Next, we performed control experiment using anti-ERK1/2 mAb to examine the expression profiles of ERK1/2 in T cells with or without co-cultivation of MAC-MΦs (Fig. 4b). The relative intensity of the ERK bands was changed during cultivation after TCR stimulation as follows: (1) ERK1 expression, MAC-MΦ (−): 0-time, 1.0; 10 min, 1.00; 20 min, 0.96; 30 min, 0.98, and MAC-MΦ (+): 0-time, 1.0; 10 min, 0.99; 20 min, 1.00; 30 min, 0.99. (2) ERK2 expression, MAC-MΦ (−): 0-time, 1.0; 10 min, 0.97; 20 min, 0.94; 30 min, 0.97, and MAC-MΦ (+): 0-time, 1.0; 10 min, 1.01; 20 min, 1.01; 30 min, 1.01. *P* values for the difference in the ERK expression profiles between T cells cultured in the presence of MAC-MΦs and T cells cultured without MAC-MΦs were insignificant as follows: ERK1, *p* = 0.61 and ERK2, *p* = 0.66. As shown in Fig. 4d, epalrestat treatment of T cells did not essentially block expression of the suppressor activity of MAC-MΦs against T-cell mitogenesis, although the suppressor effect of MAC-MΦs was slightly weakened by the pretreatment with epalrestat (*p* = 0.15 and *p* = 0.02 in the presence of 2 × 10^4^ and 4 × 10^4^ MAC-MΦs, respectively; Mann-Whitney U test). This finding suggests that the enzymatic activity itself of AR is not related to the cross-talking events between MAC-MΦ-derived suppressor signals and T-cell signal transduction pathways involving the AR-mediated axes.

Next, we attempted to identify a cellular protein of T lymphocytes, to which the AR molecule directly binds and transfers its activation signals, by performing pull-down assay using biotinylated AR. As shown in Fig. 4e, our experiments using a recombinant AR preparation, which was obtained by expressing AR molecules in insect cells transfected with human AR gene-carrying baculovirus vector, the AR molecules were found to bind to three proteins with MWs of 45, 48, and 55 kDa in the lysate of Jurkat T cells. This finding indicates that activated (phosphorylated) AR molecules transfer activating signals through molecular-to-molecular contact with certain cellular proteins, presumably those with MWs of around 50 kDa. This situation is consistent with the above observation that the enzymatic function of AR itself is not related to the intracellular transduction of MAC-MΦ-derived suppressor signals in the target T cells.

## Discussion

We previously observed that MAC-MΦ-derived suppressor signals are transmitted into target T cells via cell-to-cell contact and cause the dephosphorylation of Tyr residues of five cytosolic proteins with MWs of around 35 kDa^12^. In the present study, we newly identified one of these proteins with a MW of 36 kDa as AR. We also found that the recombinant AR preparation expressed in insect cells was constitutively phosphorylated at Tyr residues and dephosphorylated by Ba(OH)_2_ treatment. In the target T cells, intracellular expression of AR in protein levels was not altered in response to TCR stimulation or the transmission of suppressive signals from suppressor MΦs via cell contact. Therefore, it appears that AR phosphorylation and dephosphorylation are crucial for the transduction of stimulatory signals in T cells after TCR stimulation and important for the cross-talk of suppressor MΦ-derived suppressive signals with the signaling pathways for T-cell activation.

ROIs generated in response to an inflammatory stimulus are known to cause phosphorylation of intracytosolic signaling intermediates including PKC, MAPK, and IKK, which then phosphorylate IκB, inducing its degradation and translocation of NF-κB into the nucleus^32^,^33^,^34^. Subsequently, NF-κB induces the cellular expression of various genes including those encoding proinflammatory cytokines (TNF-α, IL-1β, IL-6, etc.), chemokines (MCP-1, MIP-1, etc.), and inflammatory enzymes (cyclooxygenase-2 and inducible nitric oxide synthase) that play roles in the up-regulation of immune and inflammatory reactions^35^,^36^,^37^,^38^. Notably, recent studies by Ramama and his colleagues indicated the following situations regarding the function of AR in ROI-mediated inflammatory reactions. Using different cellular (MΦs, vascular smooth muscle cells, and small airway epithelial cells) and animal models, they demonstrated that pharmacological inhibition or genetic ablation of AR prevents the AR-mediated activation of signaling kinases, thereby blocking NF-κB activation and, consequently, causing the suppression of inflammatory reactions^39^,^40^,^41^. This indicates that AR plays an important role in the cellular signaling pathways, which are linked to NF-κB-mediated inflammatory or mitogenic events in MΦs, small airway epithelial cells, and vascular smooth muscle cells in response to certain stimuli, such as LPS and TNF-α ^22−24,41^. Notably, this effect of AR is dependent upon its enzymatic activity to reduce aldehydes ^22−24,41^. They reported that AR catalyzes the reduction of lipid-aldehydes, such as 4-hydroxynonenal and glutathionyl-4-hydroxynonenal, leading to the activation of AR-mediated inflammatory signals^24^. Notably, Zeng *et al*. demonstrated that glutathionyl-1,4-dihydroxynonane, the reduced product of glutathionyl-4-hydroxynonenal due to the enzymatic action of AR, caused the sequential phosphorylation of PLC, PKC, IKK, and NF-κB-p65, leading to the activation of NF-κB and its translocation into the nucleus^18^. Moreover, Hwang *et al*. reported that another axis exists as an AR pathway which mediates JAK-STAT signaling in ischemic hearts of rats and mice^42^. According to them, global ischemia caused PKC-mediated JAK2 activation followed by STAT5 activation, and that pharmacological inhibition of AR causing a lower cytosolic NADH/NAD^+^ ratio blocked JAK2 and STAT5 activation^42^. This indicates that the function of AR in activating JAK-STAT signaling in ischemic hearts is, in part, due to changes in the cytosolic redox state.

In the present study, we found that the enzymatic activity of AR is not required for its action as a mediator of signaling pathways in T cells from the TCR to MAPK axis, because an AR inhibitor, epalrestat, did not block such T-cell signaling cascades. Since AR is commonly phosphorylated by PKC in various types of cell, such as vascular smooth muscle cells and the HL60 cell line^43^, it is suggested that phosphorylated AR molecules transmit their T-cell-stimulating signals via direct interaction with downstream molecular targets. Since PKC involving at least 10 isotypes in mammals phosphorylates Ser/Thr residues of its target proteins^44^,^45^, the target amino acid residues of AR for the PKC-mediated phosphorylation are thought to be Ser or Thr residues. However, Tyr phosphorylation of certain downstream molecules such as IκB-α also occurs via PKC-dependent signaling pathways, as demonstrated in the case of myocardinal tissue during ischemic pre-conditioning^46^. Therefore, it is also possible that PKC indirectly causes the Tyr phosphorylation of AR molecules via other signaling proteins possessing Tyr-phosphorylating activity. In any case, it is of interest to identify proteins which directly phosphorylate AR at Tyr residues.

In this context, we also found that the pharmacological inhibition of AR did not block the inhibitory activity of suppressor MΦs against T-cell mitogenesis induced by TCR-stimulating signals. Therefore, although AR is involved in T-cell signaling pathways leading to T-cell stimulation and mitogenesis via ERK1/2 activation, the enzymatic activity of AR is unnecessary for cross-talking of suppressor MΦ-derived signals with such a T-cell signaling pathway. This is consistent with the above concept that AR serves as a signaling molecule, a phosphorylated form of which directly transfers stimulatory signals to its downstream molecular targets in the T-cell signaling cascades.

In the present study, we found that co-cultivation of the target T cells with suppressor MΦs, allowing cell-to-cell contact, resulted in a marked reduction in the phosphorylation levels of ERK1/2 molecules in T cells subjected to TCR-stimulatory signals. However, the pharmacological inhibition of AR did not block the activation of ERK1/2, indicating that the enzyme activity of AR is not necessary for activation of the ERK1/2-mediated signaling axis. Meanwhile, AR up-regulates the ERK1/2-dependent signaling cascade in murine hepatocytes, and this effect is dependent upon the enzymatic activity of AR^31^. Furthermore, in human pulmonary microvascular endothelial cells, AR activity is also required for the TNF-α-induced activation of the Rho kinase/MKK4/JNK pathway but not p38 activation^15^. Taken together, it is thought that profiles of the participation of AR in signaling pathways including MAPK axes, especially ERK1/2, differ depending on the types of cell. In addition, it is suggested that the suppressive signals, which are transmitted from suppressor MΦs to the target T cells, are intracellularly transmitted through at least two signaling pathways, consisting of AR-dependent and -independent cascades.

We previously found that the transmission of suppressor signals from MAC-MΦs to target T cells via cell contact was dependent on a B7-1-like molecule (B7-1LM), which shares, in part, the same epitope with B7-1^13^. Ab blockade of CTLA-4 molecules on target T cells did not attenuate the MAC-MΦ suppressor activity, indicating that CTLA-4 does not act as a receptor for B7-1LM^13^. Separate experiments indicated that anti-PD-1 mAb-treatment of T cells abolished their susceptibility to the suppressor action of MAC-MΦs (unpublished observation). These findings suggest the possibility that suppressive signals of the suppressor MΦs are transmitted to the target T cells via PD-L1/PD-1 interactions. As reviewed by Sharpe *et al*., PD-1 transduces an inhibitory signal when engaged simultaneously with the TCR^47^,^48^. Phosphorylation of the second Tyr motif, recruits the phosphatases SHP-2 and, to a lesser extent, SHP-1 to the PD-1 cytoplasmic domain, resulting in the dephosphorylation of effector molecules activated by TCR signaling^47^. In the present study, we found that five T-cell proteins were dephosphorylated during co-cultivation with suppressor MΦs allowing cell-to-cell contact. Therefore, it is likely that the dephosphorylation of these five proteins including AR is catalyzed by SHP2 or SHP1.

It is also of interest to clarify the mechanisms by which AR transmits stimulatory signals to the downstream pathways via forming intermolecular interactions with its acceptor proteins. Thus, we attempted to identify acceptor proteins which bind to AR molecules by performing pull-down assay using biotinylated AR. Using a recombinant human AR preparation expressed in insect cells, it was found that the AR molecules directly bound to three proteins of T cells with MWs of 45, 48, and 55 kDa (Fig. 4e). This finding suggests that the MAC-MΦ-derived suppressor signal is transferred to the AR molecule via its dephosphorylation and is then directly transferred to certain proteins that bind to the AR molecule. In separate experiments based on the pull-down assay employing recombinant AR molecules, which were expressed in *Escherichia coli* cells transfected with a plasmid vector carrying the same human AR gene, we could not detect any AR-binding proteins (unpublished observations). Since protein glycosylation occurs in eukaryotic cells but not *E. coli*, these findings suggest important roles of the existence of sugar chains on the AR molecule in its protein-binding ability. Further studies are currently underway to investigate this possibility. In addition, we are attempting to identify these proteins, and their proteinaceous properties will be reported elsewhere.

## Methods

### Microorganisms

*M. avium* complex N-260 strain isolated from a patient with MAC infection was used. This MAC strain was identified as *M. intracellulare* by a DNA probe test.

### Special agents

Special agents used in this study were as follows: recombinant human AR (Wako Chemicals USA, Inc., Richmond, VA), rabbit anti-human AR Ab (Santa Cruz Biotechnology Inc., Santa Cruz, CA), horseradish peroxidase-conjugated anti-phosphotyrosine mAb (clone RC20) (BD Transduction Laboratories, Fraklin Lakes, NJ), anti-phosphoserine mAb (Calbiochem-Novabiochem Corporation, San Diego, CA), rabbit anti-p44/42 MAP kinase Ab (Cell Signaling Technology, Inc., Danvars, MA), rabbit anti-phospho-p44/42 MAP kinase Ab (Thr202/Thr204) (Cell Signaling Technology, Inc.), rat anti-mouse CD3 mAb (Serotec Ltd., Oxford, UK), hamster anti-mouse CD28 mAb (Pharmingen Co., San Diego, CA), Dynabeads^R^ mouse CD3/CD28 T cell expander (Invitrogen Dynal AS, Oslo, Norway), rat anti-mouse IL-2 mAb (R & D systems, Inc., minneapolis, MN), rat anti-mouse IFN-γ mAb (R & D systems), mouse IL-2 (R & D systems), mouse IFN-γ (R & D systems), D10.G4.1 T cell line (TIB 224; American Type Culture Collection (ATCC), Rockville, MD), epalrestat (Wako Pure Chemicals, Osaka, Japan), DL-glyceraldehyde (Wako), β-NADPH (Wako), imidazole (Wako), [^3^H]-thymidine (^3^H-TdR) (PerkinElmer Life Science Products Inc., Boston, MA).

### Medium

RPMI 1640 medium supplemented with 25 mM HEPES, 2 mM glutamine, 100 μg ml^−1^ of streptomycin, 100 units ml^−1^ of penicillin G, 5 × 10^−5^ M 2-mercaptoethanol and 5% (v/v) heat-inactivated fetal bovine serum (FBS) was used for cell culture.

### Mice

Eight to ten-week-old male BALB/c (Japan Clea Co., Osaka, Japan) were used. Animal care and experimental procedures were approved by the Animal Research Committee of Shimane University and conducted according to the regulations for Animal Experimentation at Shimane University.

### Suppressor activity of MAC-MΦs

Spleen cells (SPCs) were harvested from mice infected intravenously with 1 × 10^8^ colony forming units (CFUs) of MAC organisms at 2 to 3 weeks after infection. The resultant SPCs (MAC-SPCs) were cultured in 8 ml of 5% FBS-RPMI medium at the cell density of 5 × 10^6^ cells ml^−1^ in a 100-mm culture dish at 37 °C in a CO_2_ incubator (5% CO_2_-95% humidified air) for 2 h and washed six times with 5 ml of 2% FBS-HBSS with 1 min-vibration using Vortex mixer. Resultant adherent cells were gently scraped off with rubber policemen into 20% FBS-HBSS, centrifuged 200 × g for 5 min, finally suspended into appropriate volumes of 5% FBS-RPMI medium. This procedure usually gave more than 90% pure MΦ cultures (MAC-MΦs), with active pinocytic ability of neutral red and with phagocytic ability against latex particles. Then, 2 × 10^4^ or 4 × 10^4^ MAC-MΦs and 2.5 × 10^5^ of normal SPCs or 4.0 × 10^4^ of nylon wool column-fractionated splenic T cells were mixed and cultured in 0.2 ml of the medium containing mouse CD3/CD28 T cell expander beads (4.0 × 10^4^) (Invitrogen: anti-CD3 Ab/anti-CD28 Ab-coated beads) or 2 μg ml^−1^ Con A were poured onto the resultant MΦ cultures. SPCs and T cells were then cultivated at 37 °C in a CO_2_ incubator for 72 h and pulsed with 0.5 μCi of ^3^H-TdR (2 Ci mmol^−1^) for the final 6 to 8 h. Cells were harvested onto glass fibre filters and counted for radioactivity using a 1450 Microbeta Trilux scintillation spectrometer (Wallac Co., Turku, Finland). Suppressor activity of MAC-MΦs was calculated as:
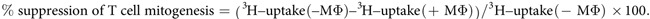


### Two-dimensional electrophoresis of T cell lysates after Cell contact with MAC-MΦs

MAC-SPCs (2 × 10^7^) were cultured in 2 ml of 5% FBS-RPMI medium in a 36-mm culture well for 2 h and washed six times with 5 ml of 2% FBS-HBSS, and the resultant adherent cells were used as MAC-MΦs. Onto the MAC-MΦ monolayer culture, 5 × 10^6^ splenic T cells were added and cultured in 2 ml of 5% FBS-RPMI medium at 37 °C for 24 h. After the culturing of T cells, allowing cell contact with MAC-MΦs, the resultant T cells (5 × 10^6^) were harvested and lysed with 0.06 ml of lysis buffer containing 10 mM Tris (pH 7.4), 150 mM NaCl, 5 mM EDTA, 1% NP-40, 1 mM Na_3_VO_4_, and 1 mM PMSF at 4 °C for 15 min. Samples were centrifuged at 13,000 × g for 5 min at 4 °C. Supernatants were collected and the protein concentration was determined with the Protein assay rapid kit (Wako). The appropriate volumes of each sample that correspond to 100 μg were subjected to two-dimensional-PAGE as follows.

A tube gel (pH 4 to 10) was used to separate the crude proteins in a 100 μl aliquot containing ampholine at the same concentrations as the tube gel by pI. The protein sample was electrophoresed using equipment for tube gel electrophoresis (NA-1313; Nippon Eido, Tokyo, Japan) at room temperature for 1.5 h at 200 V, for 9.5 h at 400 V, for 15 min at 800 V, for 15 min at 1,500 V, and then for 9.5 h at 2,500 V, with a constant voltage. The tube gel after running in the first-dimensional electrophoresis was equilibrated in 625 mM Tris-HCl (pH 6.8) containing 4% sodium dodecyl sulfate (SDS), 5% 2-mercaptoethanol, and 0.0025% bromophenol blue for 20 min (SDS buffer), and subsequently applied to the top of the SDS-PAGE gel. The tube gel was overlaid with melted 0.5% agarose containing 250 mM Tris-HCl (pH 6.8) and 0.1% SDS. The second-dimensional SDS-PAGE was then performed (using NA-1118; Nippon Eido, Tokyo, Japan) at 50 mA with a constant current. The gel after second-dimensional electrophoresis was subjected to silver staining using a Silver stain MS kit (Wako) and Western blotting for Tyr phosphorylation levels, as described below.

### In-gel protein digestion and peptide mass finger-printing

After Western blotting analysis of the gel after two-dimensional electrophoresis of the cell lysate of T lymphocytes, the silver-stained spot whose Tyr phosphorylation level was found to be decreased due to cell-to-cell contact of the T cells with MAC-MΦs (Fig. 1a), was excised from the gel and cut into small pieces. After complete destaining, the gel pieces were washed with water, and then subjected to dehydration in acetonitrile. The resultant dehydrated gel was dried, and then subjected to reduction and alkylation, and thereafter trypsin digestion using a sequencing grade-modified trypsin (Promega, Madison, WI). After overnight digestion at 37 °C, the resulting peptides were extracted into 50% acetonitrile-5% trifluoroacetic acid from the gel matrix by 30-min vortexing. The peptide samples were concentrated using ZipTip (Millipore Corp.) and then co-crystallized with 1 ml of matrix (a-cyano-4-hydroxytranscinnamic acid) on a gold-coated sample plate. Peptide mass finger-printing was performed using a PerkinElmer/PerSeptive Biosystems Voyager-DE-RP MALDI-TOF mass spectrometer, operating in delayed reflector mode at an accelerating voltage of 20 kV. A list of the corrected mass peaks was obtained as Peptide Mass Finger-printing.

### Protein identification

Protein identification was performed by searching the NCBInr database using the MASCOT search engine (http://www.matrixscience.com). The search parameters were as follows: the peptide mass tolerance was ±50 ppm, the number of missed cleavage sites was allowed to ne no more than one, the cysteine residue was modified as carbamidomethyl-cysteine, and species selection was set as a mouse. No restrictions on the protein molecular weight or *pI* value were applied.

### Western blotting

Sample proteins were separated by 10% SDS-PAGE or two-dimensional electrophoresis gels and the gels after electrophoresis were subjected to Western blotting analysis as follows. Protein bands or spots on the gels were electrotransferred to polyvinylidene fluoride (PVDF) membranes (Millipore, Bedford, MA) using 2-cyclohexyl amino-1-propane sulfonic acid (CAPS) buffer (10 mM, pH 11.0 with 10% methanol) by employing a Bio-Rad mini Trans-Blot Cell apparatus. Membranes were blocked with 1% bovine serum albumin (BSA) in TBST for 12 h at 4 °C and reacted with either horseradish peroxidase-conjugated anti-phosphotyrosine (pTyr) mAb (1:5,000 dilution), anti-phosphoserine (pSer) mAb (1:5,000 dilution), anti-AR Ab (1:750 dilution), anti-ERK1/2 (p44/42 MAP kinase) Ab (1:1,000 dilution), or anti-phospho ERK1/2 Ab (1:1,000 dilution) at room temperature for 2 h. After washing, the blots were stained with horseradish peroxidase (HRP)-conjugated protein A or HRP-conjugated anti-mouse IgM and then developed using the ECL plus kit and Western blotting detection reagents (Amersham Pharmacia Biotech Inc., Piscataway, NJ), according to the manufacturer’s instructions.

### Cytokine Assay

Concentrations of IL-2 and IFN-γ in 24-h culture fluids of T cells stimulated with anti-CD3 Ab plus anti-CD28 Ab were measured by ELISA using commercial cytokine assay kits (R & D Systems), which use rat anti-mouse IL-2 mAb and rat anti-mouse IFN-γ mAb as capture and detecting Abs, respectively. All the assay procedures were performed according to the manufacturer’s instructions. Briefly, after 2-h incubation of sample culture fluids on microtiter wells coated with individual capture Abs and subsequent washing with phosphate-buffered saline containing 0.05% Tween (Tween-PBS), the resultant wells were stained with detection Ab (biotinized goat anti-mouse IL-2 Ab, biotinized goat anti-mouse IFN-γ Ab, respectively) for 2 h, followed by washing with Tween-PBS and subsequent incubation with HRP-conjugated streptavidin for 20 min. Color development was performed using a substrate solution prepared by the manufacturer.

### Measurement for inhibitory activity of epalrestat against AR

The AR reaction mixture (1 ml) containing 100 mM Li_2_SO_4_, 10 mM DL-glyceraldehyde as a substrate, 30 μM NADPH, 0.001 units/ml AR in 135 mM Na, K-phosphate buffer (pH 7.0), with or without the addition of 1 to 10 μM epalrestat (AR inhibitor), was incubated at 30 °C for 30 min. After the addition of 0.15 ml of 0.5 N HCl, the resulting mixture was further added with 0.5 ml of 10 mM imidazole-6N NaOH, and subsequently incubated at 60 °C for 10 min. The resultant solution was measured for its fluorescence (excitation at 360 nm and emission at 460 nm) using Fluoroskan Ascent FL, Thermo Fisher Scientific, Waltham, MA).

### Screening for T cell proteins which interact with AR

Jurkat cells were treated with lysis buffer consisting of 25 mM HEPES, 150 mM NaCl, 1 mM EDTA, 1% Triton X-100, 50 mM NaF, 1% protease inhibitor cocktail, and 1 mM PMSF in ice-cold water for 30 min. After centrifugation at 13,000 × g, for 30 min, the resultant supernatant was collected and used as Jurkat cell lysate. Human AR was biotinized using an EZ-Link Micro Sulfo-NHS-Biotinylation kit (Pierce Co., Rockford, IL) according to the instructions of the manufacturer. Jurkat cell lysate (0.15 ml) containing 1 mg of protein was mixed with biotinized AR (50 μl containing 1 μg of protein) in a binding buffer (0.1% SDS-PBS). After incubation at 4 °C for 19 h, a 50-μl aliquot of streptavidin-conjugated agarose resin (Pierce) was added to the mixture and incubated at room temperature for 1 h with gentle mixing. The resultant streptavidin agarose resin was washed six times with 1 ml each of binding buffer by centrifugation (2,500 × g, 1 min) and extracted with 0.1 ml of 2× SDS sample buffer consisting of 125 mM Tris-HCl (pH 6.8), 10% 2-mercaptoethanol, 4% SDS, 10% sucrose, and 0.01% bromophenol blue by boiling for 5 min. After centrifugation, the resultant supernatant was collected and applied to 10% SDS-PAGE followed by silver staining for protein bands.

### Detection of T cell proteins that bind to AR molecules by pull-down assay

A biotinylated recombinant human AR preparation, which was prepared using EZ-Link Micro Sulfo-NHS-Biotinylation kit (PIERCE Co., Rockford, IL), was incubated with cell lysate of Jurkat T lymphocytes in the binding buffer (0.1% SDS-PBS) at 4 °C for 18 h and then incubated with streptavidin-coupled agarose resin at room temperature for 1 h. After washing with PBS, the resultant resin was boiled in the SDS buffer and then the resultant protein samples were applied to 10% SDS-PAGE followed by silver staining for the detection of the protein bands.

### Statistical analysis

Statistical analysis was performed by Bonferroni’s multiple *t*-test and Mann-Whitney U test. These statistical analyses were performed using the statistical package for social sciences (add-in software for Microsoft Excel: Excel Statistics 2008; SSRI, Tokyo, Japan) for the Windows software package. In some cases, statistical calculation for the dispersion profiles of intensity of electrophoretic bands, which were detected under the two different experimental conditions in Western blotting analysis, was performed by Two-way ANOVA (Excel, Microsoft).

## Additional Information

**How to cite this article**: Shimizu, T. *et al*. Aldose reductase participates in the downregulation of T cell functions due to suppressor macrophages. *Sci. Rep.* **6**, 21093; doi: 10.1038/srep21093 (2016).

## Figures and Tables

**Table 1:** Assignments for peptide fragments from a trypsin digest of a 36-kDa spot*.

**Experimental mass**	**Theoretical mass**	**Amino acids sequence**	**Position**
1048.47	1048.50	LIEYCHSK	196–203
1104.61	1104.63	RQDLFIVSK	70–78
1105.54	1105.50	LWCTFHDK	79–86
1241.57	1241.58	HKDYPFHAEV	307–316
1503.70	1503.68	HIDCAQVYQNEK	42–53
1586.84	1586.84	TIGVSNFNPLQIER	156–169
2217.13	2217.09	YKPAVNQIECHPYLTQEK	178–195

^*^The resulting sets of peptide masses were then used to search the NCBI database for potential matches, confirming the 36-kDa spot to be aldose reductase.
